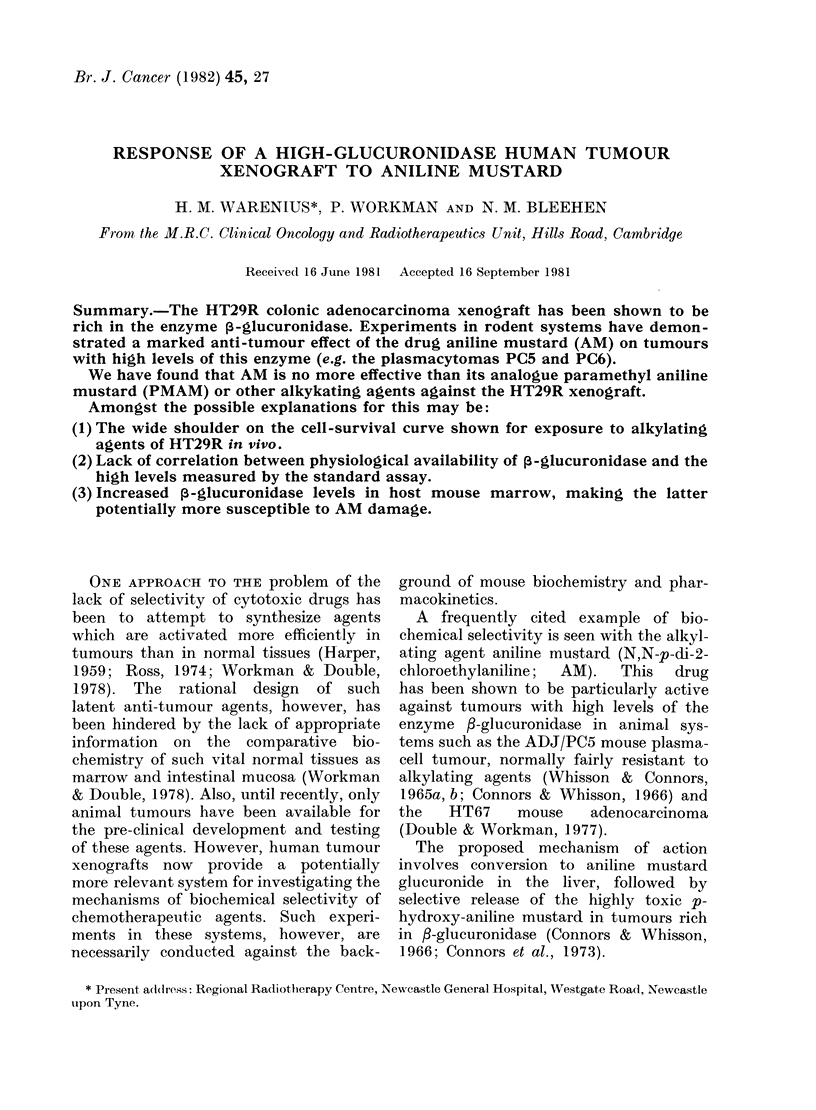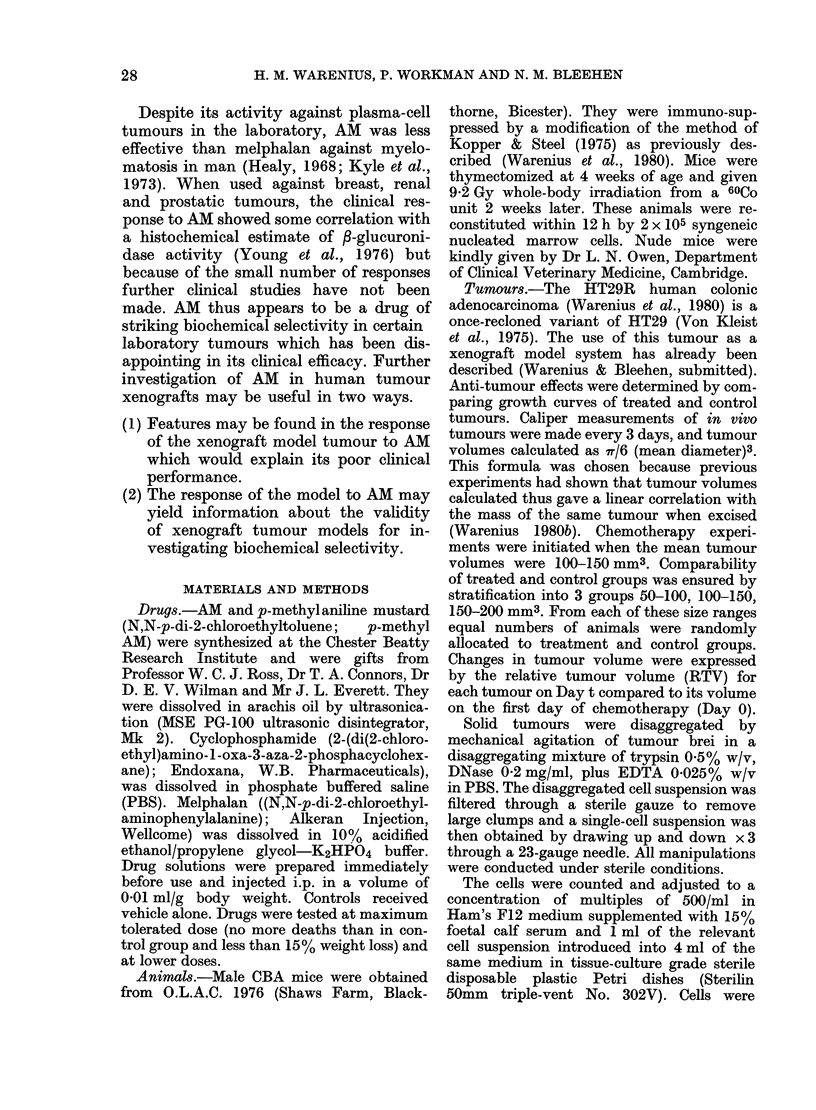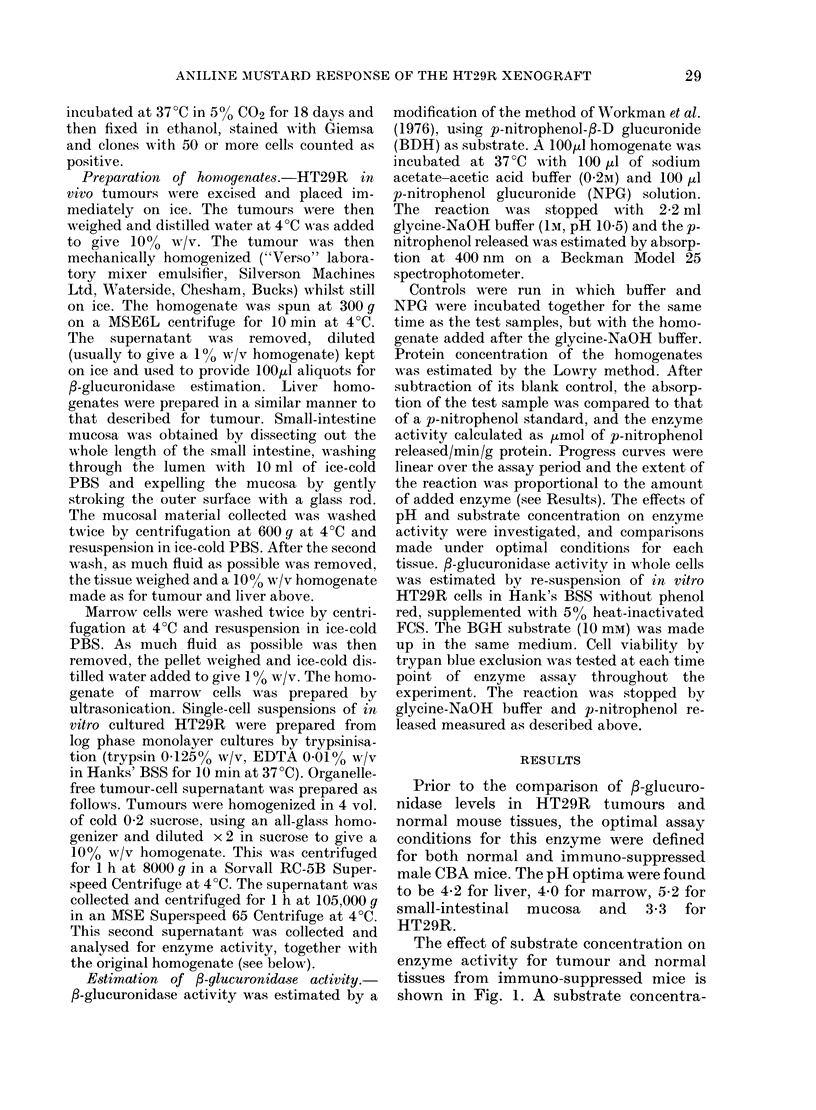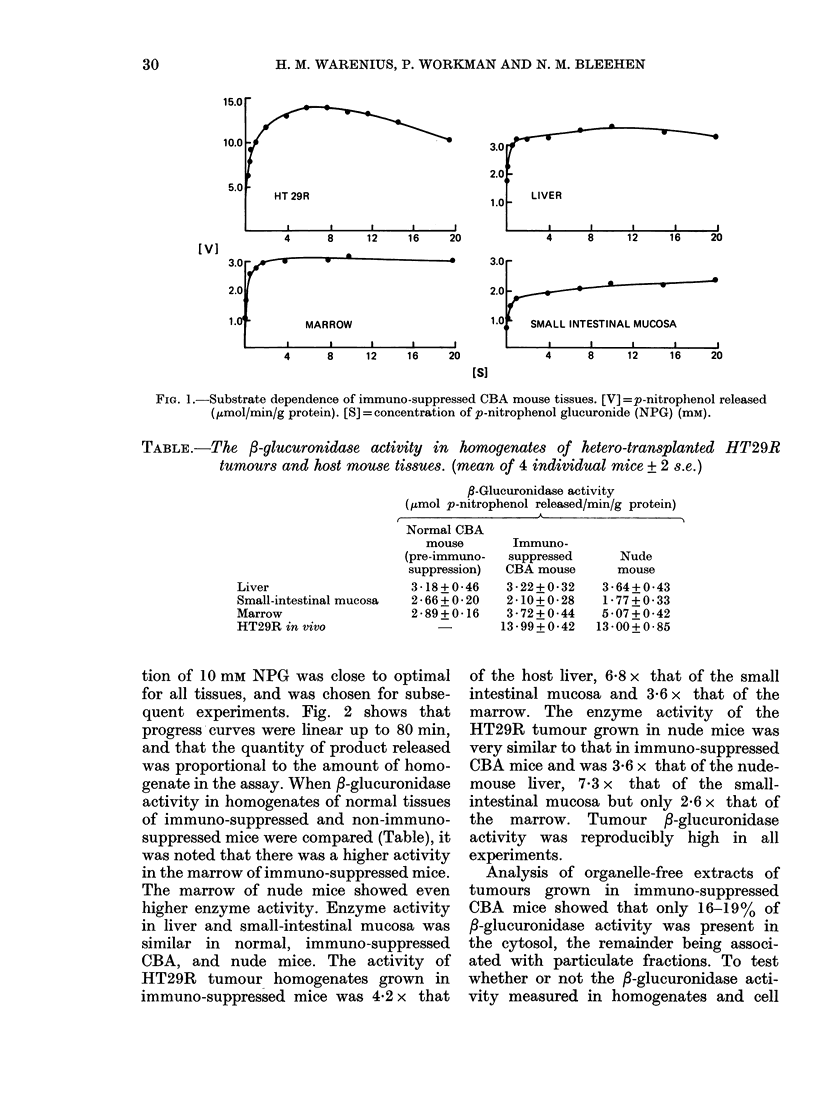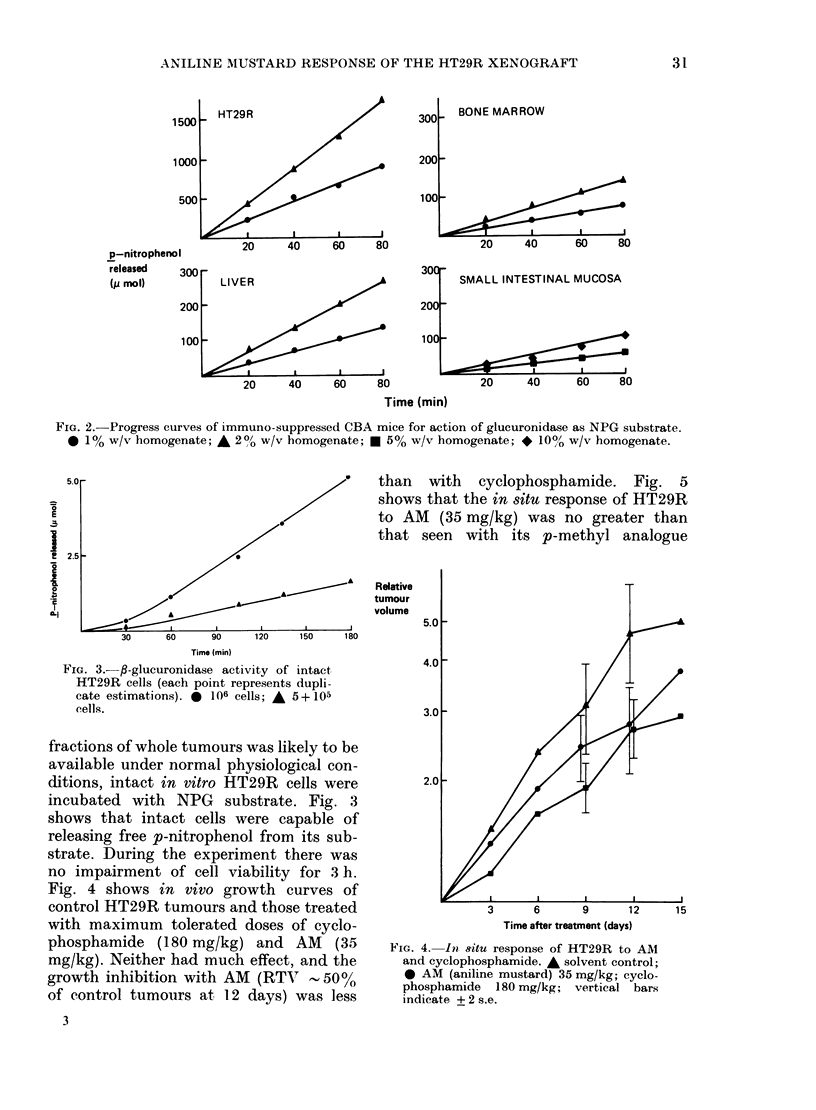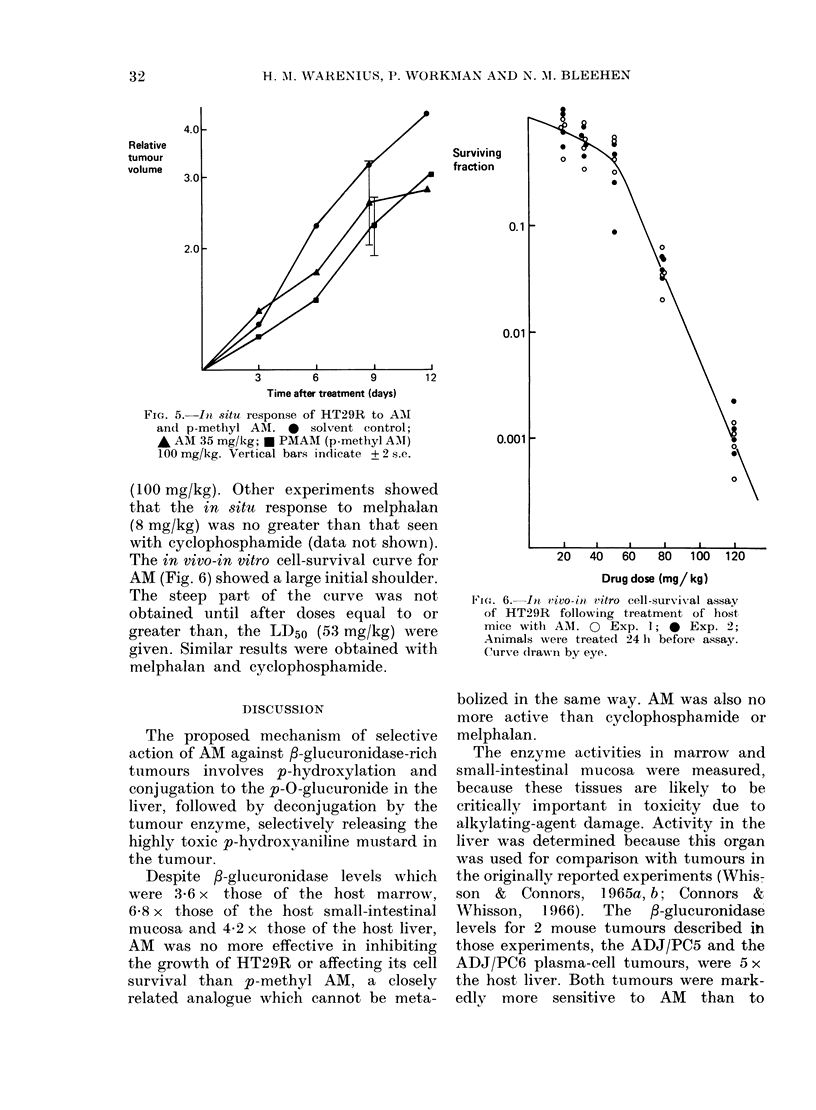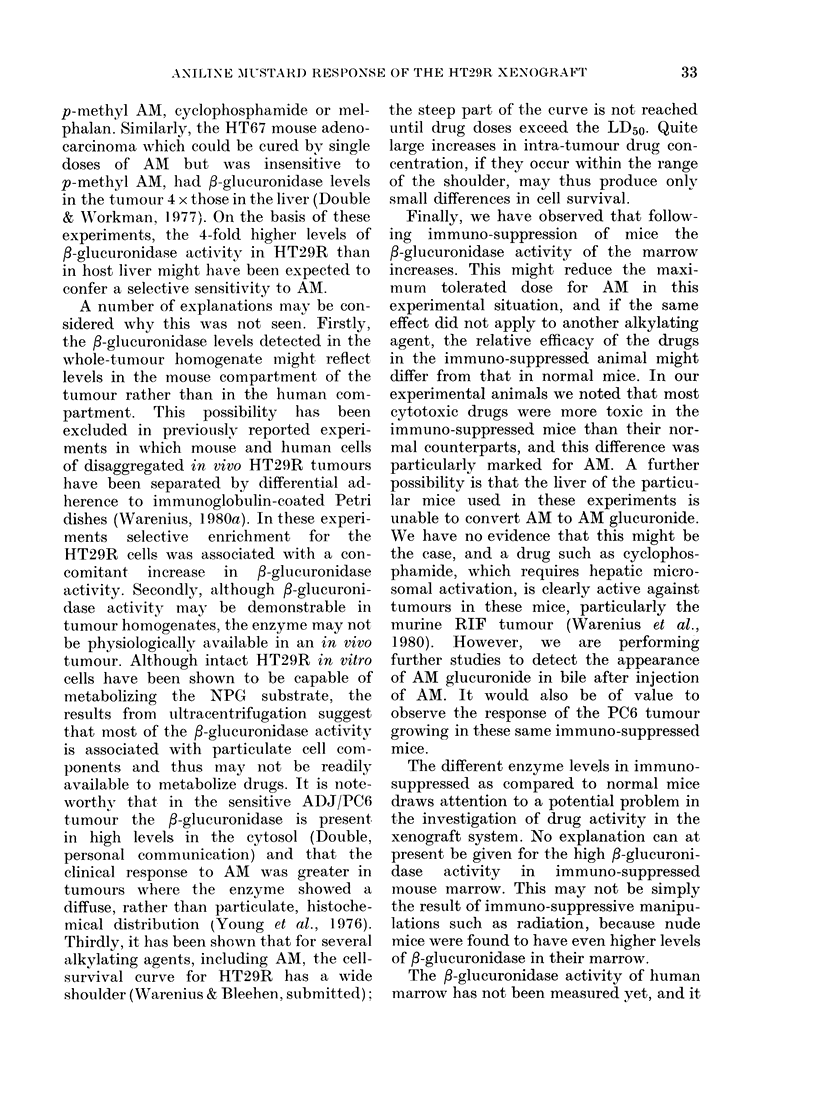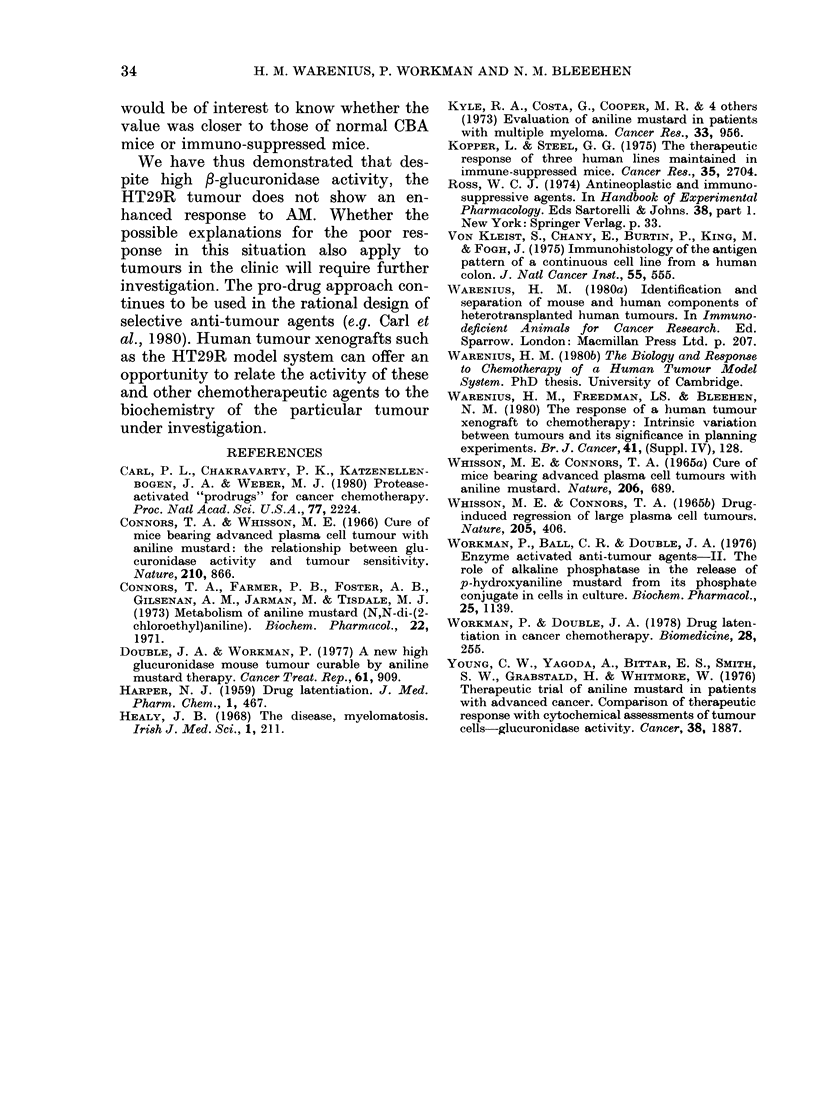# Response of a high-glucuronidase human tumour xenograft to aniline mustard.

**DOI:** 10.1038/bjc.1982.4

**Published:** 1982-01

**Authors:** H. M. Warenius, P. Workman, N. M. Bleehen

## Abstract

The HT29R colonic adenocarcinoma xenograft has been shown to be rich in the enzyme beta-glucuronidase. Experiments in rodent systems have demonstrated a marked anti-tumour effect of the drug aniline mustard (AM) on tumours with high levels of this enzyme (e.g. the plasmacytomas PC5 and PC6). We have found that AM is no more effective than its analogue paramethyl aniline mustard (PMAM) or other alkylating agents against the HT29R xenograft. Amongst the possible explanations for this may be: (1) The wide shoulder on the cell-survival curve shown for exposure to alkylating agents of HT29R in vivo. (2) Lack of correlation between physiological availability of beta-glucuronidase and the high levels measured by the standard assay. (3) Increased beta-glucuronidase levels in host mouse marrow, making the latter potentially more susceptible to AM damage.


*Br. J. Cancer* (1982) **45**, 27

### RESPONSE OF A HIGH-GLUCURONIDASE HUMAN TUMOUR XENOGRAFT TO ANILINE MUSTARD

H. M. WARENIUS*, P. WORKMAN AND N. M. BLEEHEN

*From the M.R.C. Clinical Oncology and Radiotherapeutics Unit, Hills Road, Cambridge*

Received 16 June 1981 Accepted 16 September 1981

**Summary.—The HT29R colonic adenocarcinoma xenograft has been shown to be rich in the enzyme β-glucuronidase. Experiments in rodent systems have demonstrated a marked anti-tumour effect of the drug aniline mustard (AM) on tumours with high levels of this enzyme (*e.g.* the plasmacytomas PC5 and PC6).**

**We have found that AM is no more effective than its analogue paramethyl aniline mustard (PMAM) or other alkykating agents against the HT29R xenograft.**

**Amongst the possible explanations for this may be:**

**(1) The wide shoulder on the cell-survival curve shown for exposure to alkylating agents of HT29R *in vivo*.**

**(2) Lack of correlation between physiological availability of β-glucuronidase and the high levels measured by the standard assay.**

**(3) Increased β-glucuronidase levels in host mouse marrow, making the latter potentially more susceptible to AM damage.**

ONE APPROACH TO THE problem of the lack of selectivity of cytotoxic drugs has been to attempt to synthesize agents which are activated more efficiently in tumours than in normal tissues (Harper, 1959; Ross, 1974; Workman & Double, 1978). The rational design of such latent anti-tumour agents, however, has been hindered by the lack of appropriate information on the comparative biochemistry of such vital normal tissues as marrow and intestinal mucosa (Workman & Double, 1978). Also, until recently, only animal tumours have been available for the pre-clinical development and testing of these agents. However, human tumour xenografts now provide a potentially more relevant system for investigating the mechanisms of biochemical selectivity of chemotherapeutic agents. Such experiments in these systems, however, are necessarily conducted against the background of mouse biochemistry and pharmacokinetics.

A frequently cited example of biochemical selectivity is seen with the alkylating agent aniline mustard (N,N-*p*-di-2-chloroethylaniline; AM). This drug has been shown to be particularly active against tumours with high levels of the enzyme *β*-glucuronidase in animal systems such as the ADJ/PC5 mouse plasma-cell tumour, normally fairly resistant to alkylating agents (Whisson & Connors, 1965*a*, *b*; Connors & Whisson, 1966) and the HT67 mouse adenocarcinoma (Double & Workman, 1977).

The proposed mechanism of action involves conversion to aniline mustard glucuronide in the liver, followed by selective release of the highly toxic *p*-hydroxy-aniline mustard in tumours rich in *β*-glucuronidase (Connors & Whisson, 1966; Connors *et al.*, 1973).

* Present address: Regional Radiotherapy Centre, Newcastle General Hospital, Westgate Road, Newcastle upon Tyne.

Despite its activity against plasma-cell tumours in the laboratory, AM was less effective than melphalan against myelomatosis in man (Healy, 1968; Kyle *et al.*, 1973). When used against breast, renal and prostatic tumours, the clinical response to AM showed some correlation with a histochemical estimate of β-glucuronidase activity (Young *et al.*, 1976) but because of the small number of responses further clinical studies have not been made. AM thus appears to be a drug of striking biochemical selectivity in certain laboratory tumours which has been disappointing in its clinical efficacy. Further investigation of AM in human tumour xenografts may be useful in two ways.

(1) Features may be found in the response of the xenograft model tumour to AM which would explain its poor clinical performance.

(2) The response of the model to AM may yield information about the validity of xenograft tumour models for investigating biochemical selectivity.

### MATERIALS AND METHODS

*Drugs.*—AM and *p*-methylaniline mustard (N,N-*p*-di-2-chloroethyltoluene; *p*-methyl AM) were synthesized at the Chester Beatty Research Institute and were gifts from Professor W. C. J. Ross, Dr T. A. Connors, Dr D. E. V. Wilman and Mr J. L. Everett. They were dissolved in arachis oil by ultrasonication (MSE PG-100 ultrasonic disintegrator, Mk 2). Cyclophosphamide (2-(di(2-chloroethyl)amino-1-oxa-3-aza-2-phosphacyclohexane); Endoxana, W.B. Pharmaceuticals), was dissolved in phosphate buffered saline (PBS). Melphalan ((N,N-*p*-di-2-chloroethylaminophenylalanine); Alkeran Injection, Wellcome) was dissolved in 10% acidified ethanol/propylene glycol—$K_2HPO_4$ buffer. Drug solutions were prepared immediately before use and injected i.p. in a volume of 0·01 ml/g body weight. Controls received vehicle alone. Drugs were tested at maximum tolerated dose (no more deaths than in control group and less than 15% weight loss) and at lower doses.

*Animals.*—Male CBA mice were obtained from O.L.A.C. 1976 (Shaws Farm, Blackthorne, Bicester). They were immuno-suppressed by a modification of the method of Kopper & Steel (1975) as previously described (Warenius *et al.*, 1980). Mice were thymectomized at 4 weeks of age and given 9·2 Gy whole-body irradiation from a $^{60}Co$ unit 2 weeks later. These animals were reconstituted within 12 h by $2 \times 10^5$ syngeneic nucleated marrow cells. Nude mice were kindly given by Dr L. N. Owen, Department of Clinical Veterinary Medicine, Cambridge.

*Tumours.*—The HT29R human colonic adenocarcinoma (Warenius *et al.*, 1980) is a once-recloned variant of HT29 (Von Kleist *et al.*, 1975). The use of this tumour as a xenograft model system has already been described (Warenius & Bleehen, submitted). Anti-tumour effects were determined by comparing growth curves of treated and control tumours. Caliper measurements of *in vivo* tumours were made every 3 days, and tumour volumes calculated as $\pi/6$ (mean diameter)$^3$. This formula was chosen because previous experiments had shown that tumour volumes calculated thus gave a linear correlation with the mass of the same tumour when excised (Warenius 1980*b*). Chemotherapy experiments were initiated when the mean tumour volumes were 100–150 $mm^3$. Comparability of treated and control groups was ensured by stratification into 3 groups 50–100, 100–150, 150–200 $mm^3$. From each of these size ranges equal numbers of animals were randomly allocated to treatment and control groups. Changes in tumour volume were expressed by the relative tumour volume (RTV) for each tumour on Day t compared to its volume on the first day of chemotherapy (Day 0).

Solid tumours were disaggregated by mechanical agitation of tumour brei in a disaggregating mixture of trypsin 0·5% w/v, DNase 0·2 mg/ml, plus EDTA 0·025% w/v in PBS. The disaggregated cell suspension was filtered through a sterile gauze to remove large clumps and a single-cell suspension was then obtained by drawing up and down ×3 through a 23-gauge needle. All manipulations were conducted under sterile conditions.

The cells were counted and adjusted to a concentration of multiples of 500/ml in Ham's F12 medium supplemented with 15% foetal calf serum and 1 ml of the relevant cell suspension introduced into 4 ml of the same medium in tissue-culture grade sterile disposable plastic Petri dishes (Sterilin 50mm triple-vent No. 302V). Cells were incubated at 37 °C in 5% $CO_2$ for 18 days and then fixed in ethanol, stained with Giemsa and clones with 50 or more cells counted as positive.

*Preparation of homogenates.*—HT29R *in vivo* tumours were excised and placed immediately on ice. The tumours were then weighed and distilled water at 4 °C was added to give 10% w/v. The tumour was then mechanically homogenized ("Verso" laboratory mixer emulsifier, Silverson Machines Ltd, Waterside, Chesham, Bucks) whilst still on ice. The homogenate was spun at 300 *g* on a MSE6L centrifuge for 10 min at 4 °C. The supernatant was removed, diluted (usually to give a 1% w/v homogenate) kept on ice and used to provide 100μl aliquots for β-glucuronidase estimation. Liver homogenates were prepared in a similar manner to that described for tumour. Small-intestine mucosa was obtained by dissecting out the whole length of the small intestine, washing through the lumen with 10 ml of ice-cold PBS and expelling the mucosa by gently stroking the outer surface with a glass rod. The mucosal material collected was washed twice by centrifugation at 600 *g* at 4 °C and resuspension in ice-cold PBS. After the second wash, as much fluid as possible was removed, the tissue weighed and a 10% w/v homogenate made as for tumour and liver above.

Marrow cells were washed twice by centrifugation at 4 °C and resuspension in ice-cold PBS. As much fluid as possible was then removed, the pellet weighed and ice-cold distilled water added to give 1% w/v. The homogenate of marrow cells was prepared by ultrasonication. Single-cell suspensions of *in vitro* cultured HT29R were prepared from log phase monolayer cultures by trypsinisation (trypsin 0·125% w/v, EDTA 0·01% w/v in Hanks' BSS for 10 min at 37 °C). Organelle-free tumour-cell supernatant was prepared as follows. Tumours were homogenized in 4 vol. of cold 0·2 sucrose, using an all-glass homogenizer and diluted ×2 in sucrose to give a 10% w/v homogenate. This was centrifuged for 1 h at 8000 *g* in a Sorvall RC-5B Superspeed Centrifuge at 4 °C. The supernatant was collected and centrifuged for 1 h at 105,000 *g* in an MSE Superspeed 65 Centrifuge at 4 °C. This second supernatant was collected and analysed for enzyme activity, together with the original homogenate (see below).

*Estimation of β-glucuronidase activity.*—β-glucuronidase activity was estimated by a modification of the method of Workman *et al.* (1976), using *p*-nitrophenol-β-D glucuronide (BDH) as substrate. A 100μl homogenate was incubated at 37 °C with 100 μl of sodium acetate–acetic acid buffer (0·2M) and 100 μl *p*-nitrophenol glucuronide (NPG) solution. The reaction was stopped with 2·2 ml glycine-NaOH buffer (1M, pH 10·5) and the *p*-nitrophenol released was estimated by absorption at 400 nm on a Beckman Model 25 spectrophotometer.

Controls were run in which buffer and NPG were incubated together for the same time as the test samples, but with the homogenate added after the glycine-NaOH buffer. Protein concentration of the homogenates was estimated by the Lowry method. After subtraction of its blank control, the absorption of the test sample was compared to that of a *p*-nitrophenol standard, and the enzyme activity calculated as μmol of *p*-nitrophenol released/min/g protein. Progress curves were linear over the assay period and the extent of the reaction was proportional to the amount of added enzyme (see Results). The effects of pH and substrate concentration on enzyme activity were investigated, and comparisons made under optimal conditions for each tissue. β-glucuronidase activity in whole cells was estimated by re-suspension of *in vitro* HT29R cells in Hank's BSS without phenol red, supplemented with 5% heat-inactivated FCS. The BGH substrate (10 mM) was made up in the same medium. Cell viability by trypan blue exclusion was tested at each time point of enzyme assay throughout the experiment. The reaction was stopped by glycine-NaOH buffer and *p*-nitrophenol released measured as described above.

### RESULTS

Prior to the comparison of β-glucuronidase levels in HT29R tumours and normal mouse tissues, the optimal assay conditions for this enzyme were defined for both normal and immuno-suppressed male CBA mice. The pH optima were found to be 4·2 for liver, 4·0 for marrow, 5·2 for small-intestinal mucosa and 3·3 for HT29R.

The effect of substrate concentration on enzyme activity for tumour and normal tissues from immuno-suppressed mice is shown in Fig. 1. A substrate concentra-

FIG. 1.—Substrate dependence of immuno-suppressed CBA mouse tissues. [V] = *p*-nitrophenol released (μmol/min/g protein). [S] = concentration of *p*-nitrophenol glucuronide (NPG) (mM).

TABLE.—*The β-glucuronidase activity in homogenates of hetero-transplanted HT29R tumours and host mouse tissues.* (*mean of* 4 *individual mice* ± 2 *s.e.*)

| | β-Glucuronidase activity (μmol *p*-nitrophenol released/min/g protein) | | |
|---|---|---|---|
| | Normal CBA mouse (pre-immuno-suppression) | Immuno-suppressed CBA mouse | Nude mouse |
| Liver | 3·18 ± 0·46 | 3·22 ± 0·32 | 3·64 ± 0·43 |
| Small-intestinal mucosa | 2·66 ± 0·20 | 2·10 ± 0·28 | 1·77 ± 0·33 |
| Marrow | 2·89 ± 0·16 | 3·72 ± 0·44 | 5·07 ± 0·42 |
| HT29R *in vivo* | — | 13·99 ± 0·42 | 13·00 ± 0·85 |

tion of 10 mM NPG was close to optimal for all tissues, and was chosen for subsequent experiments. Fig. 2 shows that progress curves were linear up to 80 min, and that the quantity of product released was proportional to the amount of homogenate in the assay. When β-glucuronidase activity in homogenates of normal tissues of immuno-suppressed and non-immuno-suppressed mice were compared (Table), it was noted that there was a higher activity in the marrow of immuno-suppressed mice. The marrow of nude mice showed even higher enzyme activity. Enzyme activity in liver and small-intestinal mucosa was similar in normal, immuno-suppressed CBA, and nude mice. The activity of HT29R tumour homogenates grown in immuno-suppressed mice was 4·2 × that of the host liver, 6·8 × that of the small intestinal mucosa and 3·6 × that of the marrow. The enzyme activity of the HT29R tumour grown in nude mice was very similar to that in immuno-suppressed CBA mice and was 3·6 × that of the nude-mouse liver, 7·3 × that of the small-intestinal mucosa but only 2·6 × that of the marrow. Tumour β-glucuronidase activity was reproducibly high in all experiments.

Analysis of organelle-free extracts of tumours grown in immuno-suppressed CBA mice showed that only 16–19% of β-glucuronidase activity was present in the cytosol, the remainder being associated with particulate fractions. To test whether or not the β-glucuronidase activity measured in homogenates and cell

FIG. 2.—Progress curves of immuno-suppressed CBA mice for action of glucuronidase as NPG substrate. ● 1% w/v homogenate; ▲ 2% w/v homogenate; ■ 5% w/v homogenate; ◆ 10% w/v homogenate.

FIG. 3.—β-glucuronidase activity of intact HT29R cells (each point represents duplicate estimations). ● $10^6$ cells; ▲ $5+10^5$ cells.

fractions of whole tumours was likely to be available under normal physiological conditions, intact *in vitro* HT29R cells were incubated with NPG substrate. Fig. 3 shows that intact cells were capable of releasing free *p*-nitrophenol from its substrate. During the experiment there was no impairment of cell viability for 3 h. Fig. 4 shows *in vivo* growth curves of control HT29R tumours and those treated with maximum tolerated doses of cyclophosphamide (180 mg/kg) and AM (35 mg/kg). Neither had much effect, and the growth inhibition with AM (RTV ~50% of control tumours at 12 days) was less than with cyclophosphamide. Fig. 5 shows that the *in situ* response of HT29R to AM (35 mg/kg) was no greater than that seen with its *p*-methyl analogue

FIG. 4.—*In situ* response of HT29R to AM and cyclophosphamide. ▲ solvent control; ● AM (aniline mustard) 35 mg/kg; cyclophosphamide 180 mg/kg; vertical bars indicate ±2 s.e.

FIG. 5.—*In situ* response of HT29R to AM and p-methyl AM. ● solvent control; ▲ AM 35 mg/kg; ■ PMAM (p-methyl AM) 100 mg/kg. Vertical bars indicate ±2 s.e.

FIG. 6.—*In vivo-in vitro* cell-survival assay of HT29R following treatment of host mice with AM. ○ Exp. 1; ● Exp. 2; Animals were treated 24 h before assay. Curve drawn by eye.

(100 mg/kg). Other experiments showed that the *in situ* response to melphalan (8 mg/kg) was no greater than that seen with cyclophosphamide (data not shown). The *in vivo-in vitro* cell-survival curve for AM (Fig. 6) showed a large initial shoulder. The steep part of the curve was not obtained until after doses equal to or greater than, the $LD_{50}$ (53 mg/kg) were given. Similar results were obtained with melphalan and cyclophosphamide.

### DISCUSSION

The proposed mechanism of selective action of AM against β-glucuronidase-rich tumours involves *p*-hydroxylation and conjugation to the *p*-O-glucuronide in the liver, followed by deconjugation by the tumour enzyme, selectively releasing the highly toxic *p*-hydroxyaniline mustard in the tumour.

Despite β-glucuronidase levels which were 3·6× those of the host marrow, 6·8× those of the host small-intestinal mucosa and 4·2× those of the host liver, AM was no more effective in inhibiting the growth of HT29R or affecting its cell survival than *p*-methyl AM, a closely related analogue which cannot be metabolized in the same way. AM was also no more active than cyclophosphamide or melphalan.

The enzyme activities in marrow and small-intestinal mucosa were measured, because these tissues are likely to be critically important in toxicity due to alkylating-agent damage. Activity in the liver was determined because this organ was used for comparison with tumours in the originally reported experiments (Whisson & Connors, 1965*a*, *b*; Connors & Whisson, 1966). The β-glucuronidase levels for 2 mouse tumours described in those experiments, the ADJ/PC5 and the ADJ/PC6 plasma-cell tumours, were 5× the host liver. Both tumours were markedly more sensitive to AM than to

*p*-methyl AM, cyclophosphamide or melphalan. Similarly, the HT67 mouse adenocarcinoma which could be cured by single doses of AM but was insensitive to *p*-methyl AM, had β-glucuronidase levels in the tumour 4 × those in the liver (Double & Workman, 1977). On the basis of these experiments, the 4-fold higher levels of β-glucuronidase activity in HT29R than in host liver might have been expected to confer a selective sensitivity to AM.

A number of explanations may be considered why this was not seen. Firstly, the β-glucuronidase levels detected in the whole-tumour homogenate might reflect levels in the mouse compartment of the tumour rather than in the human compartment. This possibility has been excluded in previously reported experiments in which mouse and human cells of disaggregated *in vivo* HT29R tumours have been separated by differential adherence to immunoglobulin-coated Petri dishes (Warenius, 1980*a*). In these experiments selective enrichment for the HT29R cells was associated with a concomitant increase in β-glucuronidase activity. Secondly, although β-glucuronidase activity may be demonstrable in tumour homogenates, the enzyme may not be physiologically available in an *in vivo* tumour. Although intact HT29R *in vitro* cells have been shown to be capable of metabolizing the NPG substrate, the results from ultracentrifugation suggest that most of the β-glucuronidase activity is associated with particulate cell components and thus may not be readily available to metabolize drugs. It is noteworthy that in the sensitive ADJ/PC6 tumour the β-glucuronidase is present in high levels in the cytosol (Double, personal communication) and that the clinical response to AM was greater in tumours where the enzyme showed a diffuse, rather than particulate, histochemical distribution (Young *et al.*, 1976). Thirdly, it has been shown that for several alkylating agents, including AM, the cell-survival curve for HT29R has a wide shoulder (Warenius & Bleehen, submitted); the steep part of the curve is not reached until drug doses exceed the $LD_{50}$. Quite large increases in intra-tumour drug concentration, if they occur within the range of the shoulder, may thus produce only small differences in cell survival.

Finally, we have observed that following immuno-suppression of mice the β-glucuronidase activity of the marrow increases. This might reduce the maximum tolerated dose for AM in this experimental situation, and if the same effect did not apply to another alkylating agent, the relative efficacy of the drugs in the immuno-suppressed animal might differ from that in normal mice. In our experimental animals we noted that most cytotoxic drugs were more toxic in the immuno-suppressed mice than their normal counterparts, and this difference was particularly marked for AM. A further possibility is that the liver of the particular mice used in these experiments is unable to convert AM to AM glucuronide. We have no evidence that this might be the case, and a drug such as cyclophosphamide, which requires hepatic microsomal activation, is clearly active against tumours in these mice, particularly the murine RIF tumour (Warenius *et al.*, 1980). However, we are performing further studies to detect the appearance of AM glucuronide in bile after injection of AM. It would also be of value to observe the response of the PC6 tumour growing in these same immuno-suppressed mice.

The different enzyme levels in immuno-suppressed as compared to normal mice draws attention to a potential problem in the investigation of drug activity in the xenograft system. No explanation can at present be given for the high β-glucuronidase activity in immuno-suppressed mouse marrow. This may not be simply the result of immuno-suppressive manipulations such as radiation, because nude mice were found to have even higher levels of β-glucuronidase in their marrow.

The β-glucuronidase activity of human marrow has not been measured yet, and it would be of interest to know whether the value was closer to those of normal CBA mice or immuno-suppressed mice.

We have thus demonstrated that despite high $\beta$-glucuronidase activity, the HT29R tumour does not show an enhanced response to AM. Whether the possible explanations for the poor response in this situation also apply to tumours in the clinic will require further investigation. The pro-drug approach continues to be used in the rational design of selective anti-tumour agents (*e.g.* Carl *et al.*, 1980). Human tumour xenografts such as the HT29R model system can offer an opportunity to relate the activity of these and other chemotherapeutic agents to the biochemistry of the particular tumour under investigation.

### REFERENCES

CARL, P. L., CHAKRAVARTY, P. K., KATZENELLENBOGEN, J. A. & WEBER, M. J. (1980) Protease-activated "prodrugs" for cancer chemotherapy. *Proc. Natl Acad. Sci. U.S.A.*, **77**, 2224.

CONNORS, T. A. & WHISSON, M. E. (1966) Cure of mice bearing advanced plasma cell tumour with aniline mustard: the relationship between glucuronidase activity and tumour sensitivity. *Nature*, **210**, 866.

CONNORS, T. A., FARMER, P. B., FOSTER, A. B., GILSENAN, A. M., JARMAN, M. & TISDALE, M. J. (1973) Metabolism of aniline mustard (N,N-di-(2-chloroethyl)aniline). *Biochem. Pharmacol.*, **22**, 1971.

DOUBLE, J. A. & WORKMAN, P. (1977) A new high glucuronidase mouse tumour curable by aniline mustard therapy. *Cancer Treat. Rep.*, **61**, 909.

HARPER, N. J. (1959) Drug latentiation. *J. Med. Pharm. Chem.*, **1**, 467.

HEALY, J. B. (1968) The disease, myelomatosis. *Irish J. Med. Sci.*, **1**, 211.

KYLE, R. A., COSTA, G., COOPER, M. R. & 4 others (1973) Evaluation of aniline mustard in patients with multiple myeloma. *Cancer Res.*, **33**, 956.

KOPPER, L. & STEEL, G. G. (1975) The therapeutic response of three human lines maintained in immune-suppressed mice. *Cancer Res.*, **35**, 2704.

ROSS, W. C. J. (1974) Antineoplastic and immunosuppressive agents. In *Handbook of Experimental Pharmacology*. Eds Sartorelli & Johns. **38**, part 1. New York: Springer Verlag. p. 33.

VON KLEIST, S., CHANY, E., BURTIN, P., KING, M. & FOGH, J. (1975) Immunohistology of the antigen pattern of a continuous cell line from a human colon. *J. Natl Cancer Inst.*, **55**, 555.

WARENIUS, H. M. (1980*a*) Identification and separation of mouse and human components of heterotransplanted human tumours. In *Immunodeficient Animals for Cancer Research*. Ed. Sparrow. London: Macmillan Press Ltd. p. 207.

WARENIUS, H. M. (1980*b*) *The Biology and Response to Chemotherapy of a Human Tumour Model System*. PhD thesis. University of Cambridge.

WARENIUS, H. M., FREEDMAN, LS. & BLEEHEN, N. M. (1980) The response of a human tumour xenograft to chemotherapy: Intrinsic variation between tumours and its significance in planning experiments. *Br. J. Cancer*, **41**, (Suppl. IV), 128.

WHISSON, M. E. & CONNORS, T. A. (1965*a*) Cure of mice bearing advanced plasma cell tumours with aniline mustard. *Nature*, **206**, 689.

WHISSON, M. E. & CONNORS, T. A. (1965*b*) Drug-induced regression of large plasma cell tumours. *Nature*, **205**, 406.

WORKMAN, P., BALL, C. R. & DOUBLE, J. A. (1976) Enzyme activated anti-tumour agents—II. The role of alkaline phosphatase in the release of *p*-hydroxyaniline mustard from its phosphate conjugate in cells in culture. *Biochem. Pharmacol.*, **25**, 1139.

WORKMAN, P. & DOUBLE, J. A. (1978) Drug latentiation in cancer chemotherapy. *Biomedicine*, **28**, 255.

YOUNG, C. W., YAGODA, A., BITTAR, E. S., SMITH, S. W., GRABSTALD, H. & WHITMORE, W. (1976) Therapeutic trial of aniline mustard in patients with advanced cancer. Comparison of therapeutic response with cytochemical assessments of tumour cells—glucuronidase activity. *Cancer*, **38**, 1887.